# Response to Penetrance estimates of hereditary cancers in a population setting using UK Biobank data

**DOI:** 10.1038/s44276-023-00031-9

**Published:** 2024-03-15

**Authors:** Leigh Jackson, Michael N. Weedon, Harry D. Green, Bethan Mallabar-Rimmer, Jamie W. Harrison, Andrew R. Wood, Katherine S. Ruth, Jess Tyrrell, Caroline F. Wright

**Affiliations:** Institute of Biomedical and Clinical Science, University of Exeter College of Medicine and Health, Royal Devon & Exeter Hospital, RILD Building, Barrack Road, Exeter, EX2 5DW UK

**replying to** R. Manchanda et al. BJC Reports 10.1038/s44276-023-00021-x (2024)

A recent commentary by Manchanda et al. has made a number of criticisms of Jackson et al. [1] that we feel compelled to address. These observations seem to fall broadly into three categories: critique of our methodological approach, discussion of the suitability of using UK Biobank data, and concerns about interpretation.

Before we address these points, it is important to note the final sentence of the author’s commentary, which states that family history (FH) should be included in a comprehensive risk assessment of cancer susceptibility genes (*BRCA1, BRCA2* and Lynch syndrome genes). On this we agree, and in fact, the sole point made in the interpretation section of the abstract of our paper was “family history should be considered when counselling patients on the risks and benefits of potential follow-up care.” Nowhere in the paper do we state or intend to imply that those without a FH are at no risk or should not be referred for surveillance and clinical follow-up. Indeed, we clearly show an elevated risk amongst pathogenic variants carriers in UK Biobank, and deliberately use the term ‘less-elevated’ to describe the group lacking FH to make clear there is still a significant risk of carrying pathogenic variants regardless of FH. Reduced penetrance in clinically unselected populations has now been widely observed across a range of monogenic conditions [2–5].

The criticism of our methodology, we believe, is misplaced. The authors incorrectly state that individuals who had cancer before the study’s commencement are not included in the analysis, and it is therefore not appropriate to consider the participants at risk from birth. However, UK Biobank contains linked Cancer Registry Data dating back to the early 1970s (30–40 years prior to recruitment), and indeed over 5600 participants were diagnosed with breast cancer prior to inclusion in the study, with the earliest diagnosis age of 18 years old. Given our focus on adult-onset cancers, and the mean age in UK Biobank at the latest cancer registry data freeze of 69 years (56 years at recruitment), with >50 years of cancer registry data, it is reasonable to conclude we have sufficient data on included individuals to consider them in our analyses as included from birth. The authors’ point about consistent hazard assumptions over time is fair and was addressed in the discussion of our paper: “Cox model assumes proportional hazards over time and linearity, which may not hold true for inherited cancer cohorts and may impact the estimates derived”. We regret any confusion caused by the inclusion of lifetime risk estimates from NICE guidance in Figure 2—labelled as such and intended simply as a reference for comparison.

A related point is made about this cohort being under-representative for those having cancer prior to recruitment and this influencing their decision to enrol. On this point, we entirely agree and estimate the potential impact of this survivor bias in our discussion. The calculated 11% depletion in female BRCA variant carries compared to males represents a decent proxy for the magnitude of this missing cohort and, even assuming all missing carriers had cancer, does not substantially alter our penetrance estimates. We also state, “this bias will have the effect of removing very highly penetrant variants from the cohort, which is likely to deflate penetrance estimates”. We also agree with the remaining criticisms of the UK Biobank cohort and clearly stated these in our discussion, with the following caveat: “UK Biobank is also not a representative population cohort, due to recognised recruitment biases and so these estimates are likely to represent a lower bound.” Throughout the discussion we were clear that the findings of our study were not intended to represent a real-world penetrance estimate but a lower bound, making the distinction that FH is an important discriminator.

It remains clear that in a cohort of individuals similar to that in UK Biobank, the penetrance of cancer, whether modelled or directly observed, is lower than existing estimates derived from clinical cohorts. (Directly observed penetrance of pathogenic *BRCA1* and *BRCA2* variants to age 60, when only considering individuals who were over 60 at the time of the cancer registry data freeze in 2021, was 33.9% and 24.4%, respectively. The penetrance in older age groups is also lower than previously reported, but we are less confident in the estimates since the number of carriers is small.) The commentary reproaches us for ignoring larger datasets, yet the study cited in support of this comment is referenced in our paper and does not represent what the authors purport it to. Although Kuchenbaecker et al. [6] do indeed have more pathogenic variant carriers than are present in UK Biobank, individuals were recruited through various consortia explicitly enriched for cancers and familial cases. It is not a population cohort comparable to UK Biobank, but a clinical cohort derived from a number of studies primarily recruiting through cancer genetic clinics. Therefore, while crucial for estimating risks amongst families and individuals eligible for clinical genetic testing, it is open to ascertainment bias towards enriching for highly penetrant variants—exactly the bias that we are trying to balance with our study. Ascertainment bias is influenced by different and often unmeasured modifiers, both known and unknown, genetic and environmental, which collectively affect whether or not a pathogenic genetic variant is penetrant in an individual. It is therefore important to estimate penetrance across a range of different studies with different, potentially opposing, ascertainment biases. Replication in other population cohorts in future will be essential.

The primary motivation for our study was the interpretation of secondary or incidental genetic findings. Our concerns are around the risk faced by individuals identified outside of existing clinical pathways, in whom there is little suspicion of cancer (familial or otherwise). Imagine, for example, the healthy parent of a child with severe developmental delay, who has had their genome sequenced in an effort to diagnose the cause of the child’s condition. When a pathogenic *BRCA1* variant is discovered incidentally in the mother, how should she be counselled? What is the most representative cohort from which to derive risk estimates for her? The risks for individuals and their families ascertained as a result of their cancers are well studied, and excellent datasets exist to guide counselling decisions for this group. However, the same is not true of those discovering their genotype with no other clinical or familial indications for increased cancer risk. These individuals also suffer significant distress and uncertainty. Whilst we can neither endorse nor control everything written in the mainstream media relating to our work, our aim was to reduce unnecessary harm to such individuals through potential overdiagnosis and overtreatment.

Although UK Biobank may not be the perfect cohort in which to determine the penetrance of pathogenic variants in clinically unselected individuals, it is nonetheless the largest and most representative currently available and as such, the results are worthy of reporting.

## Data Availability

No datasets were generated or analysed during the current study.
